# Design and implementation of an efficient single layer five input majority voter gate in quantum-dot cellular automata

**DOI:** 10.1186/s40064-016-2220-7

**Published:** 2016-05-17

**Authors:** Ali Newaz Bahar, Sajjad Waheed

**Affiliations:** Department of Information and Communication Technology, Mawlana Bhashani Science and Technology University, Tangail, 1902 Bangladesh

**Keywords:** Quantum-dot cellular automata (QCA), Five-input majority gate (MV_5_), QCA full-adder (FA), QCADesigner, Expandable MV

## Abstract

The fundamental logical element of a quantum-dot cellular automata (QCA) circuit is majority voter gate (MV). The efficiency of a QCA circuit is depends on the efficiency of the MV. This paper presents an efficient single layer five-input majority voter gate (MV_5_). The structure of proposed MV_5_ is very simple and easy to implement in any logical circuit. This proposed MV_5_ reduce number of cells and use conventional QCA cells. However, using MV_5_ a multilayer 1-bit full-adder (FA) is designed. The functional accuracy of the proposed MV_5_ and FA are confirmed by QCADesigner a well-known QCA layout design and verification tools. Furthermore, the power dissipation of proposed circuits are estimated, which shows that those circuits dissipate extremely small amount of energy and suitable for reversible computing. The simulation outcomes demonstrate the superiority of the proposed circuit.

## Background

Now a day’s, CMOS technology is approaching its physical boundary and facing earnest challenges by designing perpetually incrementing frequencies and downscaling of computational devices. This technology has found many complication like high leakage current, high power consumption, high lithography cost, low density problem and limitation of speed in GHz range. Therefore, to overcome the deficiencies an extensive research on nanotechnologies must be taken into consideration. A report of ITRS (International Technology Road 2013) shows a road map of future computing technologies. Quantum-dot cellular automata (Lent et al. 1993; Orlov et al. 1997) is one of the promising alternative technologies that proffers an innovative approach and has exhibited ultra low power, extreme speed and highly dense digital devise designing capabilities. In addition, QCA based memory unit, reversible logic and arithmetic logic circuit have been considered in several studies (Kim et al. 2007; Navi et al. 2010; Hänninen and Takala 2010; Hashemi et al. 2012; Qanbari and Sabbaghi-Nadooshan 2013; Kianpour and Sabbaghi-Nadooshan 2014; Sayedsalehi et al. 2015; Angizi et al. 2015; Bahar et al. 2015).

The rudimentary element of QCA circuit is a majority gate (MV); digital operation can be employed by using MV. MV characterizes and determines the function value based on majority verdict (Oya et al. 2003). Up to now, most QCA circuits have been investigated and designed only by means of 3-input majority gates (MV_3_). However, if these circuits are constructed using 5-input majority gates (MV_5_), they would be optimized in cell counts, area and complexity.

To reveal the effectiveness of proposed MV_5_, a QCA full-adder has been designed using proposed MV_5_. Results reveal the superiority of proposed FA in terms of latency, cell counts and area to other previous designs (Tougaw and Lent 1994; Wang et al. 2003; Zhang et al. 2004; Azghadi et al. 2007; Cho and Swartzlander 2007, 2009).

## Proposed five-input majority gate

MV_5_ is a cell arrangement which includes five input cells, one output and some device cells. The logic function of MV_5_ can be presented as Eq. (1), where the inputs are labeled as A, B, C, D and E respectively. The truth table of the MV_5_ is shown in Table 1.

**Table 1:** Truth table of MV_5_ based on sum of inputs

Σ (A, B, C, D, E)	MV (A, B, C, D, E)
0	0
1	0
2	0
3	1
4	1
5	1

1 $$MV \left( {A, B, C, D, E} \right) = ABC\,+\,ABD\,+\,ABE\,+\,ACD\,+\,ACE\,+\,ADE\,+\,BCD\,+\,BCE\,+\,BDE\,+\,CDE$$

**Fig. 1:**
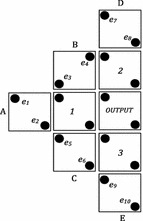
Proposed 5-input majority gate

The proposed design of MV_5_ is shown in Fig. 1. In this design, *A*, *B*, *C*, *D* and *E* are labeled as inputs and the output cell is labeled as *OUTPUT*. Additionally, three middle cells are labeled 1, 2 and 3. Polarization of input cells are fixed and middle cells and output cell are free to change. Here, cell “A” has an impact on all the middle cells. Similarly, cell “B”, cell “C” cell “D” and cell “E” also have an impact on all the middle cells. These impacts are propagated to the output cell and construct the MV_5_ output, efficiently. The propose MV_5_ requires only nine cells and uses conventional QCA cells to implement.

## Physical proofs

**Fig. 2:**
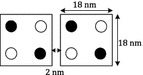
Two rectangular QCA cell

To carry out the physical proofs, the below postulates are considered:

- All cells are alike and the distance of end to end of each cell is 18 nm.
- The space between two neighbor cells is 2 nm shown in Fig. 2.

The proposed MV_5_ has approximately 32 distinct input states; we should verify all the input condition to validate the accuracy of the gate. In this paper, only one state (*A* = 1, *B* = 0, *C* = *D* = *E* = 1) has been considered for verification. Similarly, other states can be verified too. For a fixed input MV_5_, the five input cells polarization are remain unchanged; only the intermediary cells and the output cell are subject to be changed to their polarization according to the input cells. Here, the proposed MV_5_ have three intermediary cells and one output cell those are labeled as 1, 2, 3 and *OUTPUT* respectively shown in Fig. 1.

A structure is said to be stable, when the QCA cells are assembled with their minimum potential energies. The potential energy between two different cell electrons can be computed using the Eq. (2) (Halliday and Resnick 2004; McDermott 1984; Halloun and Hestenes 1985). Here, *U* is potential energy; a fixed colon is *k*, *q*_1_ and *q*_2_ are electric charges, and the distance between two electric charges is *r*. The total potential energy of a given structure is “*U*_*T*_” and that can be calculated using Eq. (3).

2 $$U = \frac{{kq_{1} q_{2} }}{r}$$

3 $$U_{T} = \mathop \sum \limits_{i = 1}^{n} U_{i}$$

**Fig. 3:**
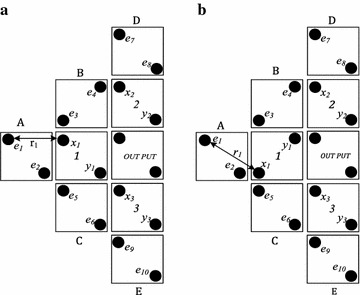
Five-input majority gate **a** cell-1 polarization *P* = −1, **b** cell-1 polarization *P* = +1

For, finding the stable structure, one needs to calculate the potential energy *U*_*i*_ for each middle cell. Here, cell 1 has two different polarization state; polarization *P* = +1 and *P* = −1 shown in Fig. 3.

Now, considering state 3 (a); here the potential energy for cell 1 *U*_*T*_ is the summation of potential energies of both x and y electrons. Potential energy for x and y electrons are the total energy exist between each electron (*e*_1_, *e*_2_, *e*_3_, *e*_4_, *e*_5_, *e*_6_, *e*_7_, *e*_*8*_, *e*_*9*_, and *e*_10_) with electron x and y respectively, which is calculated using Eq. (2). Finally, using Eq. (3) total potential energy for “cell 1” can be calculated. Similarly, potential energy of “cell 1” for state 3.3 (b) can be calculated. The necessary calculations for finding the total potential energies of structure (a) and structure (b) are given below:

**Table:** 

Figure 3a (For electron x)	Figure 3a (For electron y)
$U_{1} = \frac{A}{{r_{1} }} = \frac{{23.04 \times 10^{ - 29} }}{{20 \times 10^{ - 9} }} \approx 1.15 \times 10^{ - 20} J$ $U_{2} = \frac{A}{{r_{2} }} = \frac{{23.04 \times 10^{ - 29} }}{{18.11 \times 10^{ - 9} }} \approx 1.27 \times 10^{ - 20} J$ $U_{3} = \frac{A}{{r_{3} }} = \frac{{23.04 \times 10^{ - 29} }}{{2 \times 10^{ - 9} }} \approx 11.52 \times 10^{ - 20} J$ $U_{4} = \frac{A}{{r_{4} }} = \frac{{23.04 \times 10^{ - 29} }}{{26.91 \times 10^{ - 9} }} \approx 0.86 \times 10^{ - 20} J$ $U_{5} = \frac{A}{{r_{5} }} = \frac{{23.04 \times 10^{ - 29} }}{{20 \times 10^{ - 9} }} \approx 1.15 \times 10^{ - 20} J$ $U_{6} = \frac{A}{{r_{6} }} = \frac{{23.04 \times 10^{ - 29} }}{{42.04 \times 10^{ - 9} }} \approx 0.55 \times 10^{ - 20} J$ $U_{7} = \frac{A}{{r_{7} }} = \frac{{23.04 \times 10^{ - 29} }}{{44.72 \times 10^{ - 9} }} \approx 0.52 \times 10^{ - 20} J$ $U_{8} = \frac{A}{{r_{8} }} = \frac{{23.04 \times 10^{ - 29} }}{{43.91 \times 10^{ - 9} }} \approx 0.53 \times 10^{ - 20} J$ $U_{9} = \frac{A}{{r_{9} }} = \frac{{23.04 \times 10^{ - 29} }}{{44.72 \times 10^{ - 9} }} \approx 0.52 \times 10^{ - 20} J$ $U_{10} = \frac{A}{{r_{10} }} = \frac{{23.04 \times 10^{ - 29} }}{{69.34 \times 10^{ - 9} }} \approx 0.33 \times 10^{ - 20} J$ $U_{{T_{{}} x_{1} }}^{ - } = \sum\nolimits_{i = 1}^{10} {U_{i} } = 18.38 \times 10^{ - 20} J$	$U_{1} = \frac{A}{{r_{1} }} = \frac{{23.04 \times 10^{ - 29} }}{{42.04 \times 10^{ - 9} }} \approx 0.55 \times 10^{ - 20} J$ $U_{2} = \frac{A}{{r_{2} }} = \frac{{23.04 \times 10^{ - 29} }}{{20 \times 10^{ - 9} }} \approx 1.15 \times 10^{ - 20} J$ $U_{3} = \frac{A}{{r_{3} }} = \frac{{23.04 \times 10^{ - 29} }}{{26.91 \times 10^{ - 9} }} \approx 0.86 \times 10^{ - 20} J$ $U_{4} = \frac{A}{{r_{4} }} = \frac{{23.04 \times 10^{ - 29} }}{{38 \times 10^{ - 9} }} \approx 0.61 \times 10^{ - 20} J$ $U_{5} = \frac{A}{{r_{5} }} = \frac{{23.04 \times 10^{ - 29} }}{{18.11 \times 10^{ - 9} }} \approx 1.27 \times 10^{ - 20} J$ $U_{6} = \frac{A}{{r_{6} }} = \frac{{23.04 \times 10^{ - 29} }}{{20 \times 10^{ - 9} }} \approx 1.15 \times 10^{ - 20} J$ $U_{7} = \frac{A}{{r_{7} }} = \frac{{23.04 \times 10^{ - 29} }}{{58.03 \times 10^{ - 9} }} \approx 0.40 \times 10^{ - 20} J$ $U_{8} = \frac{A}{{r_{8} }} = \frac{{23.04 \times 10^{ - 29} }}{{44.72 \times 10^{ - 9} }} \approx 0.52 \times 10^{ - 20} J$ $U_{9} = \frac{A}{{r_{9} }} = \frac{{23.04 \times 10^{ - 29} }}{{22.09 \times 10^{ - 9} }} \approx 1.04 \times 10^{ - 20} J$ $U_{10} = \frac{A}{{r_{10} }} = \frac{{23.04 \times 10^{ - 29} }}{{44.72 \times 10^{ - 9} }} \approx 0.52 \times 10^{ - 20} J$ $U_{{T_{{y_{1} }} }}^{ - } = \sum\nolimits_{i = 1}^{10} {U_{i} } = 8.05 \times 10^{ - 20} J$

Total potential energy of Fig. 3a is

$$U_{{T_{1} }}^{ - } = 26.42 \times 10^{ - 20} J$$

Similarly, the total potential energy for Fig. 3b can be calculated and it is

$$U_{{T_{1} }}^{ + } = 36.33 \times 10^{ - 20} J$$

With comparison of the achieved results, the electrons in cell 1 are located in state (a) is more stable because it has the lower potential energy than state (b). Similar the potential energy for cell 2 and cell 3 can be calculated and the final results are mentioned as.

$$U_{{_{2} }}^{ - } = 23.39 \times 10^{ - 20} J\quad U_{{T_{2} }}^{ + } = 23.86 \times 10^{ - 20} J$$

$$U_{{T_{3} }}^{ - } = 14.35 \times 10^{ - 20} J\quad U_{{T_{3} }}^{ + } = 33.38 \times 10^{ - 20} J$$

## Proposed QCA full-adder

**Fig. 4:**
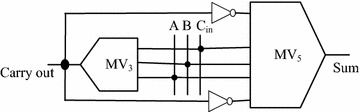
Schematic diagram of QCA full-adder

The proposed MV_5_ is implemented by designing an efficient QCA full-adder. The schematic diagram of newly proposed QCA full-adder is shown in Fig. 4.

This full-adder is designed using the planar designing concept. The proposed FA has been implemented using 2-inverters and 2-MVs. In comparison with the earlier FA (Azghadi et al. 2007), it has an extra inverter gate. The structure of proposed MV_5_, it would be easier to employ 2-inverters rather than 1-inverter and some wires for transmitting the inverted signal to other part. The proposed QCA FA is simple in structure and easy to construct. In this design, at first the carry value is calculated and then takes its inversion value and uses this value as an input of the MV_5_ gates.

## Power dissipation of proposed QCA full-adder

The power dissipates from a single cell depends on the rate of change of the clock and the tunneling energy. The power dissipation of a QCA circuit in a single clock phase can be simply calculated by adding the power dissipated by each majority gate and inverter (Liu et al. 2012).

Using Hamming distance (HD) power dissipation of a QCA circuit can be estimated. Power dissipation is depends on HD between input cells to inverter cells as well as HD between majority voter gates (Liu et al. 2012). For an inverter when the input is changed from 0 → 0 or 1 → 1. In this case the HD will be 0, and the power dissipation by inverter at *γ* = 0.25 *E*_*k*_ and *T* = 2.0 *K* is 0.8 meV whereas for *γ* = 1.0 *E*_*k*_, it is 8.0 meV (Liu et al. 2012). If the input is changed from 0 → 1 or 1 → 0, in this case the HD will be 1 and the power dissipation by the inverter is 28.4 meV, where *T* = 2.0 *K* and *γ* = 0.25 *E*_*k*_. For majority gate, power dissipation is minimum, when the inputs are changed from 000 → 000 i.e. HD is 0, and the power dissipation is maximum when polarization of all inputs are changed i.e. input polarization are changed from 000 → 111 i.e. HD is 3. The power dissipation by the majority voter gate for HD 0 and 3 are 0.8 and 41.0 meV respectively, where *γ* = 0.25 *E*_*k*_ and *T* = 2.0 *K* (Liu et al. 2012).

By using Hamming distance based methodology described in (Liu et al. 2012), the power dissipated by the proposed MV_5_ and 1-bit QCA full-adder is estimated and the results are shown in Table 2.

**Table 2:** Power dissipation of proposed five input majority gate and QCA full adder

	Power dissipation at *T* = 2.0 K			
	*γ* = 0.25 *E*_*k*_ (meV)	*γ* = 0.50 *E*_*k*_ (meV)	*γ* = 0.75 *E*_*k*_ (meV)	*γ* = 1.0 *E*_*k*_ (meV)
Five input majority gate	75.3	77.8	80.6	84.5
QCA full-adder	125.9	129.1	136.8	145.4

## Simulations and results comparison

**Fig. 5:**
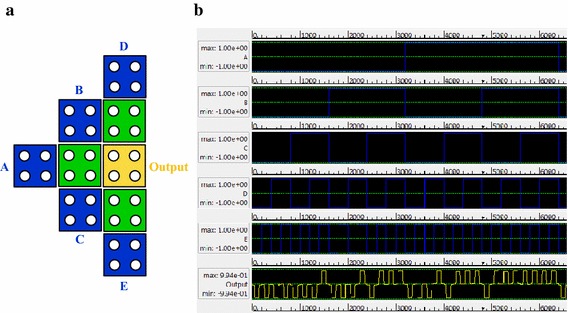
Simulated **a** circuit layout and **b** input–output wave form of proposed MV_5_

The proposed MV_5_ and FA have been simulated and verified using QCADesigner (Walus et al. 2003, 2004) only tools for QCA layout design and verification. In these simulations, both bi-stable and coherence vector engines have been employed to simulate. In both simulations identical outputs are obtained which confirm the correctness of the proposed designs. The simulated circuit layout and simulated output of proposed MV_5_ are shown in Fig. 5.

**Fig. 6:**
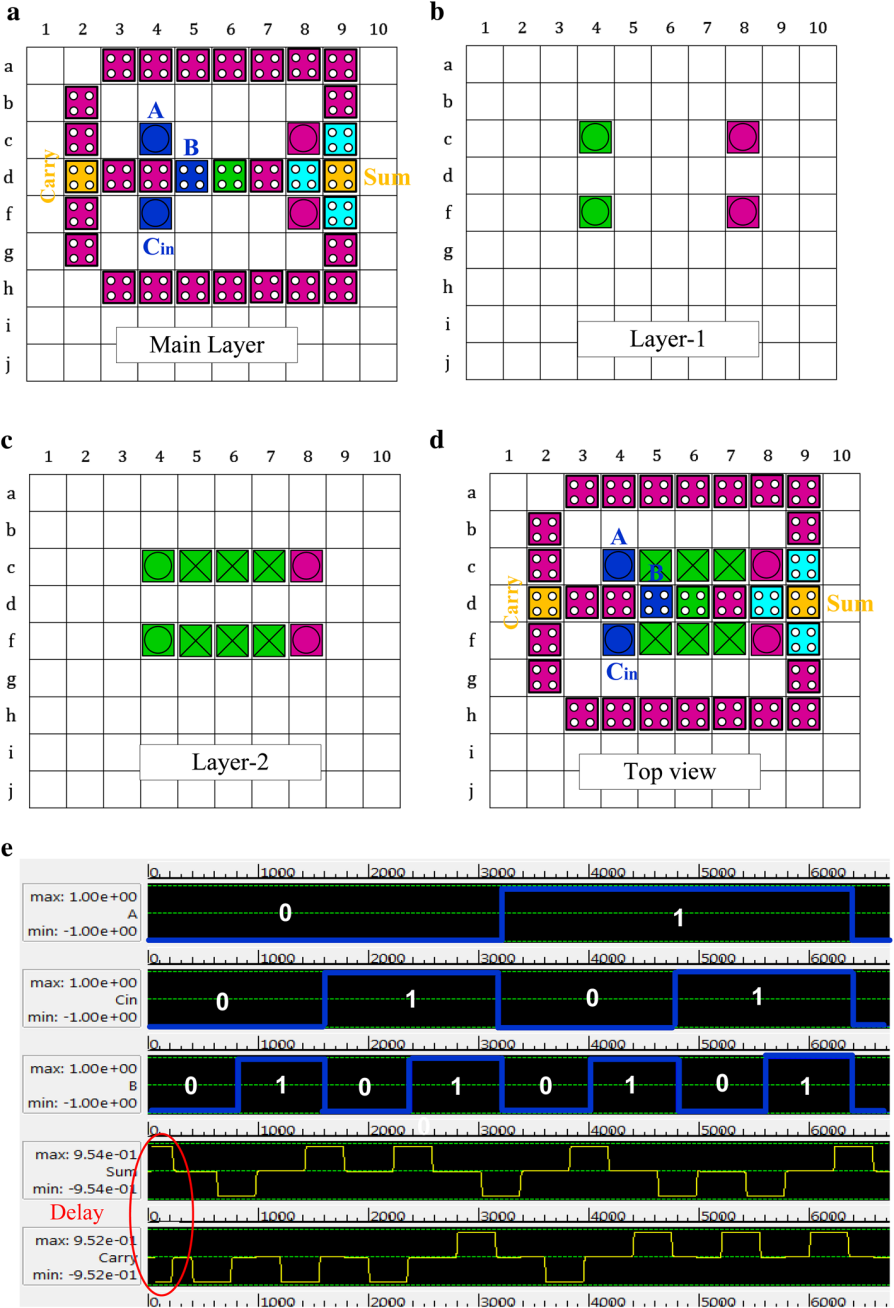
Simulated circuit layout of proposed full-adder **a** main layer, **b** layer-1, **c** layer-2, **d** *top view* of the adder, **e** simulation result

The proposed QCA full-adder is designed in three layers illustrated in Fig. 6. The main layer contains 34 cells, second layer contains 4 cells and the third layer contains 10 cells. Finally, it requires 48 cells and 3 clock phases to produce exact outputs (Sum and Carry).

Using QCADesigner, complexity, time delay and area consumption of QCA circuits can easily be calculated (Walus et al. 2003). Table 3 demonstrates a concise comparison between the proposed QCA FA and the earlier FA (Vetteth et al. 2002; Wang et al. 2003; Zhang et al. 2005; Cho and Swartzlander 2007; Kim et al. 2007; Cho and Swartzlander 2007, 2009; Navi et al. 2010; Hänninen and Takala 2010; Hashemi et al. 2012; Qanbari and Sabbaghi-Nadooshan 2013) in terms of complexity, area and time delay. Here, complexity indicates the number of cell is used to design the FA. Similarly, the area represents the total covered area of the corresponding FA in micro meter. The “Latency” indicates the number of clock zone used. It also indicates the time delay of the circuit.

**Table 3:** Comparison of QCA full-adders in terms of gate count, area and latency

Full adder	Type of full adder	Complexity (cells)	Area (μm^2^)	Latency (clock cycle)
FA [1]	Coplanar QCA FA (Vetteth et al. 2002)	292	0.62	3.5
FA [2]	Robust QCA FA (Kim et al. 2007)	220	0.36	3
FA [3]	Coplanar QCA FA (Wang et al. 2003)	145	0.17	1.25
FA [4]	Type-I QCA FA (Cho and Swartzlander 2007)	135	0.14	1.25
FA [5]	Multilayer QCA FA (Zhang et al. 2005)	108	0.10	1
FA [6]	The Robust QCA FA (Hänninen and Takala 2010)	102	0.10	2
FA [7]	Type-II QCA FA (Cho 2006; Azghadi et al. 2007)	86	0.10	0.75
FA [8]	Robust QCA FA (Hashemi et al. 2012)	79	0.05	1.25
FA [9]	Multilayer QCA FA (Navi et al. 2010)	73	0.04	0.75
FA [10]	Multilayer QCA FA (Qanbari and Sabbaghi-Nadooshan 2013)	63	0.05	0.75
	Proposed full adder	48	0.03	0.75

**Fig. 7:**
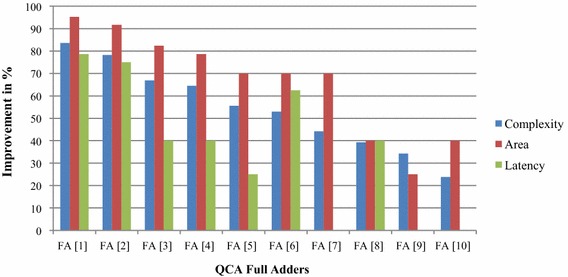
A comparative analysis of proposed full-adder with previous

It is clear that the new QCA full-adder dominates all the previous designs (Vetteth et al. 2002; Wang et al. 2003; Zhang et al. 2005; Cho and Swartzlander 2007; Kim et al. 2007; Cho and Swartzlander 2007, 2009; Navi et al. 2010; Hänninen and Takala 2010; Hashemi et al. 2012; Qanbari and Sabbaghi-Nadooshan 2013) in terms of covered area and number of cell count. It leads to a very dense structure and has the same time delay with the previous best designs (Qanbari and Sabbaghi-Nadooshan 2013). According to the bar chat shown in Fig. 7, the proposed FA leads to around 95.17 % improvement in area and 83.6 percent improvement in cell complexity in comparison to the QCA FA designed using 3-input majority gates and inverters in (Vetteth et al. 2002). This FA also, leads to around 40 % improvement in area and 23.8 % improvement in cell complexity compared to the best QCA FA designed using previous MV_5_ and inverters (Qanbari and Sabbaghi-Nadooshan 2013).

## Reliability of proposed QCA circuits

**Fig. 8:**
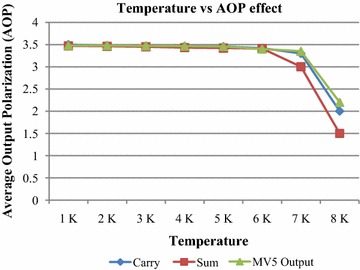
The effect of temperature on average output polarization (AOP) of proposed MV_5_ and QCA FA

The temperature effect on the output cell’s polarization of proposed MV_5_ and QCA FA are observed. The output cell’s polarization is taken at different temperature using QCADesigner tool. The average output polarization (AOP) for each output cell is calculated from (Pudi and Sridharan 2011) and shown in Fig. 8. The proposed circuit works efficiently in temperature range of 1–6 K, and the AOP for each output cell is changed very little in this range. When the temperature is above 6 K, the AOP is dropped drastically, which results incorrect outputs.

## Conclusion

A new flexible 5-input majority gate and a new efficient full-adder have been presented. The proposed MV_5_ has been implemented in one layer and using nine QCA cells only. To validate the correctness and effectiveness of the proposed MV_5_ a QCA FA has been presented. Moreover the estimation of power dissipation by the proposed QCA full-adder circuits illustrates that the proposed QCA FA is highly energy efficient circuit. The proposed FA has a considerable improvement in comparison to the previous FAs in terms of covered area, number of cells and has a similar time delay to the previous best FA.

## References

[CR1] Angizi S, Moaiyeri MH, Farrokhi S, Navi K, Bagherzadeh N (2015). Designing quantum-dot cellular automata counters with energy consumption analysis. Microprocess Microsyst.

[CR2] Azghadi MR, Kavehei O, Navi K (2007). A novel design for quantum-dot cellular automata cells and full adders. J Appl Sci.

[CR3] Bahar AN, Waheed S, Hossain N (2015). A new approach of presenting reversible logic gate in nanoscale. SpringerPlus.

[CR4] Cho H (2006) Adder and multiplier design and analysis in quantum-dot cellular automata. PhD dissertation, Faculty of the Graduate School, University of Texas, Austin

[CR5] Cho H, Swartzlander EE (2007). Adder designs and analyses for quantum-dot cellular automata. IEEE Trans Nanotechnol.

[CR6] Cho H, Swartzlander EE (2009). Adder and multiplier design in quantum-dot cellular automata. IEEE Trans Comput.

[CR7] Halliday D, Resnick A (2004). Fundamentals of physics, part 1, chapters 3–6.

[CR8] Halloun IA, Hestenes D (1985). Common sense concepts about motion. Am J Phys.

[CR9] Hänninen I, Takala J (2010). Binary adders on quantum-dot cellular automata. Sci J Circ Syst Signal Process.

[CR10] Hashemi S, Tehrani M, Navi K (2012). An efficient quantum-dot cellular automata full-adder. Sci Res Essays.

[CR11] International Technology Road map for Semiconductors (ITRS) (2013) International Roadmap Committee. http://www.itrs.net Accessed 11 Oct 2015

[CR12] Kianpour M, Sabbaghi-Nadooshan R (2014). A conventional design and simulation for CLB implementation of an FPGA quantum-dot cellular automata. Microprocess Microsyst.

[CR13] Kim K, Wu K, Karri R (2007). The robust QCA adder designs using composable QCA building blocks. IEEE Trans Comput Aided Des Integr Circ Syst.

[CR14] Lent CS, Tougaw PD, Porod W (1993). Bistable saturation in coupled quantum dots for quantum cellular automata. Appl Phys Lett.

[CR15] Liu W, Srivastava S, Lu L, O’Neill M, Swartzlander EE (2012). Are QCA cryptographic circuits resistant to power analysis attack?. IEEE Trans Nanotechnol.

[CR16] McDermott LC (1984). Research on conceptual understanding in mechanics. Phys Today.

[CR17] Navi K, Farazkish R, Sayedsalehi S, Azghadi MR (2010). A new quantum-dot cellular automata full-adder. Microelectr J.

[CR18] Orlov A, Amlani I, Bernstein G, Lent C, Snider G (1997). Realization of a functional cell for quantum-dot cellular automata. Science.

[CR19] Oya T, Asai T, Fukui T, Amemiya Y (2003). A majority-logic device using an irreversible single-electron box. IEEE Trans Nanotechnol.

[CR20] Pudi V, Sridharan K (2011). Efficient design of a hybrid adder in quantum-dot cellular automata. IEEE Trans Very Large Scale Integr Syst.

[CR21] Qanbari M, Sabbaghi-Nadooshan R (2013). Two Novel quantum-dot cellular automata full adders. J Eng.

[CR22] Sayedsalehi S, Azghadi MR, Angizi S, Navi K (2015). Restoring and non-restoring array divider designs in quantum-dot cellular automata. Inf Sci.

[CR23] Tougaw PD, Lent CS (1994). Logical devices implemented using quantum cellular automata. J Appl Phys.

[CR24] Vetteth A, Walus K, Dimitrov VS, Jullien GA (2002) Quantum-dot cellular automata carry-look-ahead adder and barrel shifter. In: IEEE Emerging Telecommunications Technologies Conference 2–4

[CR25] Walus K, Dimitrov V, Jullien GA, Miller WC (2003) QCADesigner: a CAD tool for an emerging nano-technology. In: Micronet Annual Workshop, pp 292–294

[CR26] Walus K, Dysart TJ, Jullien GA, Budiman RA (2004). QCADesigner: a rapid design and simulation tool for quantum-dot cellular automata. IEEE Trans Nanotechnol.

[CR27] Wang W, Walus K, Jullien GA (2003) Quantum-dot cellular automata adders. In: 2003 third IEEE conference on nanotechnology, NANO 2003, IEEE, vol 1, pp 461–464

[CR28] Zhang R, Walus K, Wang W, Jullien GA (2004). A method of majority logic reduction for quantum cellular automata. IEEE Trans Nanotechnol.

[CR29] Zhang R, Walus K, Wang W, Jullien GA (2005) Performance comparison of quantum-dot cellular automata adders. In: IEEE international symposium on circuits and systems, ISCAS 2005, IEEE, vol 03, pp 2522–2526

